# *Mycobacterium tuberculosis*-Induced Upregulation of the COX-2/mPGES-1 Pathway in Human Macrophages Is Abrogated by Sulfasalazine

**DOI:** 10.3389/fimmu.2022.849583

**Published:** 2022-05-19

**Authors:** Wenfei Wang, Yuping Ning, Yejun Wang, Guofang Deng, Simona Pace, Stefanie A. Barth, Christian Menge, Kehong Zhang, Youchao Dai, Yi Cai, Xinchun Chen, Oliver Werz

**Affiliations:** ^1^ Department of Pharmaceutical/Medicinal Chemistry, Institute of Pharmacy, Friedrich-Schiller-University, Jena, Germany; ^2^ Guangdong Provincial Key Laboratory of Regional Immunity and Diseases, Department of Pathogen Biology, Shenzhen University School of Medicine, Shenzhen, China; ^3^ Guangdong Key Laboratory for Emerging Infectious Diseases, Shenzhen Third People’s Hospital, Shenzhen, China; ^4^ Friedrich-Loeffler-Institut/Federal Research Institute for Animal Health, Institute of Molecular Pathogenesis, Jena, Germany

**Keywords:** Mycobacterium tuberculosis, cyclooxygenase, sulfasalazine, macrophages, prostaglandin E_2_

## Abstract

Macrophages are the primary human host cells of intracellular *Mycobacterium tuberculosis* (*M.tb*) infection, where the magnitude of inflammatory reactions is crucial for determining the outcome of infection. Previously, we showed that the anti-inflammatory drug sulfasalazine (SASP) significantly reduced the *M.tb* bactericidal burden and histopathological inflammation in mice. Here, we asked which genes in human inflammatory macrophages are affected upon infection with *M.tb* and how would potential changes impact the functional state of macrophages. We used a flow cytometry sorting system which can distinguish the dead and alive states of *M.tb* harbored in human monocyte-derived macrophages (MDM). We found that the expression of cyclooxygenase-2 and microsomal prostaglandin E_2_ synthase (mPGES)-1 increased significantly in tagRFP^+^ MDM which were infected with alive *M.tb*. After exposure of polarized M1-MDM to *M.tb* (H37Rv strain)-conditioned medium (MTB-CM) or to the *M.tb*-derived 19-kD antigen, the production of PGE_2_ and pro-inflammatory cytokines increased 3- to 4-fold. Upon treatment of M1-MDM with SASP, the MTB-CM-induced expression of COX-2 and the release of COX products and cytokines decreased. Elevation of PGE_2_ in M1-MDM upon MTB-CM stimulation and modulation by SASP correlated with the activation of the NF-κB pathway. Together, infection of human macrophages by *M.tb* strongly induces COX-2 and mPGES-1 expression along with massive PGE_2_ formation which is abrogated by the anti-inflammatory drug SASP.

## Introduction

Tuberculosis (TB) represents a devastating communicable disease caused by *Mycobacterium tuberculosis* (*M.tb*) that is responsible for about 10 million infections and 1.4 million human deaths every year (1). The fate of macrophages plays a vital role in the pathogenesis, and also in the host defense against *M.tb* (2). Micro-environmental signals are critical for macrophage differentiation and polarization, with the pro-inflammatory M1 and the pro-resolving M2 phenotypes being the two extreme subtypes (3, 4). Normally, classically activated M1 macrophages are induced by microbial stimuli which are also key effectors of the host response against intracellular bacteria. However, accumulating evidence suggests that both the pathogen and the invaded host cell exhibit marked phenotypic heterogeneity during the infection process (5, 6). Dissecting the key events or molecules which determine the phenotypic heterogeneity are important for development of precise medicine against intracellular pathogens.

Inflammation is a self-protective process of the host during which the immune system defends against exterior stimuli (7). Host immunity and the inflammatory response are also closely linked with the outcome of tuberculosis infection (8). Elevated levels of lipid mediators (LM) such as prostanoids are frequently associated with inflammation. Thus, release of arachidonic acid (AA) from membrane phospholipids leads to production of prostaglandin (PG)E_2_ catalyzed by cyclooxygenases (COX) and connected PGE_2_ synthases. Macrophages up-regulate the inducible COX-2 isoform in response to inflammatory signals and are thus major producers of PGE_2_ and other prostanoids (9). Highly expressed COX-2 protein in malignant tissue is also associated with poor prognosis and outcome in cancer disease (10, 11). PGE_2_ acts through four distinct receptors (EP1-4) of which EP2 seems to play a critical role in host susceptibility to *M.tb* infection (12, 13). However, whether or not COX-2 expression is modulated in immune cells in human TB disease is still elusive.

In order to identify genes that are affected in human inflammatory macrophages upon *M.tb* infection, we established a flow cytometry sorting system to accurately isolate different bacteria-exposed human monocyte-derived macrophage (MDM) subgroups which were then collected for further gene expression profiling. Furthermore, a single-cell RNA-sequencing library-construction method was established to solve the problem of RNA extraction and sequencing from small numbers of subgroup cells. Our data show that expression of COX-2 and microsomal PGE_2_ synthase (mPGES)-1 are significantly increased in macrophages with alive *M.tb* bacteria. Comprehensive LM profiling in these human MDM revealed most potent elevations of PGE_2_ that increased 3- to 4-fold upon stimulation with *M.tb* (H37Rv strain)-conditioned medium (MTB-CM) or with the *M.tb* 19-kD antigen. The anti-inflammatory drug sulfasalazine (SASP), approved by the FDA for clinical use, exerts inhibitory effects against tumor growth, tumor cell invasion, and metastasis in many types of cancers (14–16). Previously, we found that mice treated with SASP displayed significantly reduced *M.tb* bacterial load in the lung, lymph node, spleen, and liver (17). As SASP is an anti-inflammatory drug and PGE_2_ is pro-inflammatory, acting as a switch between bacterial clearance and acute disease, we hypothesized that SASP may potentially interfere with the COX-2/PGE_2_ pathway and thus prevent severe inflammation caused by excessive PGE_2_ during *M.tb* infection.

## Materials and Methods

### Materials

Deuterated and non-deuterated LM standards for ultra-performance liquid chromatography-tandem mass spectrometry (UPLC-MS-MS) quantification were purchased from Cayman Chemical/Biomol GmbH (Hamburg, Germany). SASP and all reagents were obtained from Sigma-Aldrich (Taufkirchen, Germany) unless mentioned otherwise.

### Human Subjects and Samples

Plasma samples were collected from human subjects at the Shenzhen Third People’s Hospital. The experimental protocol was approved by the ethical committee of Shenzhen Third People’s Hospital. Diagnosis of active TB in these subjects was based on chest radiography, clinical symptoms, and microscopy for acid fast bacilli (AFB), sputum and/or BALF *M.tb* culture and the response to anti-TB chemotherapy. Healthy controls (HC) with normal chest radiographic outcome and without clinical history of TB were recruited. *M.tb*-specific interferon-γ release assays (IGRA) were used to differentiate individuals with latent TB infection (LTBI) from HC without an infection. The non-TB lung diseases group included patients with pneumonia (PN). Plasma samples were stored at −80°C. Unless otherwise indicated, the samples from patients were collected prior to initiation of antibiotic treatment.

### Cell Isolation and Polarization of Human MDM

Leukocyte concentrates from freshly withdrawn peripheral blood of male and female healthy adult volunteers were provided by the Institute of Transfusion Medicine at the University Hospital Jena (Jena, Germany). The experimental protocol was approved by the ethical committee of the University Hospital Jena. All methods were performed in accordance with the relevant guidelines and regulations. Polymorphonuclear leukocytes (PMNL), peripheral blood monocytic cells (PBMC) and platelets were separated using dextran sedimentation, followed by centrifugation on lymphocyte separation medium (Histopaque-1077) as reported elsewhere by us (18). For isolation of monocytes, PBMC were resuspended in RPMI 1640 containing 10% (v/v) heat-inactivated fetal calf serum (FCS), 100 U/ml penicillin and 100 µg/ml streptomycin in cell culture flasks (Greiner Bio-One, Frickenhausen, Germany) for 1.5 h at 37°C and 5% CO_2_ for adherence of monocytes. For differentiation of the monocytes to MDM and further polarization towards an M1-like phenotype, published criteria were used (19). M1-MDM were generated by incubating monocytes with 20 ng/ml GM-CSF (PeproTech, Hamburg, Germany) for 6 d in RPMI 1640 supplemented with 10% FCS, 2 mmol/L glutamine (Biochrom/Merck, Berlin, Germany), and penicillin–streptomycin (Biochrom/Merck) to obtain M0_GM-CSF_MDM (briefly “MDM”), followed by treatment with 100 ng/ml lipopolysaccharide (LPS) and 20 ng/ml IFNγ (PeproTech) to obtain pro-inflammatory M1-like MDM. Routinely, MDM were polarized for 48 h unless stated otherwise.

### Mycobacterial Infection

Live-dead reporter *M.tb* strain H37Ra constitutively expresses Emerald and a tetracycline inducible tgRFP. Bacteria were cultured in 0.5 mg/ml Hygromycin 7H9 media (BD Biosciences, San Jose, CA, USA) at 37°C in log phase, washed, sonicated, and passed through 0.45 μm Millex filter to obtain a single cell suspension. For experiments involving induction of tgRFP fluorescence expression in transcriptionally active bacteria, anhydrotetracycline (200 ng/ml) was added for 24 h.

MDM were infected with *M.tb* strain H37Ra at a MOI of 5. After 4 h incubation, non-internalized bacteria were washed away from MDM with PBS and incubation with RPMI 1640 medium. For colony-forming unit (CFU) assays, cells were incubated for 72 h and then lysed with 0.1% SDS. Cell lysates were diluted, plated on 7H11 Middlebrooks agar plates, and incubated at 37°C and 5% CO_2_. CFU were counted 3 to 4 weeks later. For gene expression assay, cells were harvested at 24 h after infection and subjected to RNA isolation. For flow cytometry, cells were harvested at 72 h and were subjected to fluorescence-activated cell sorting (FACS) for further analysis. In some experiments, cells were pretreated with SASP for 1 h.

### *M.tb* (H37Rv)-Conditioned Medium (MTB-CM)

*M.tb* (H37Rv) was inoculated in Middlebrook 7H9 broth with glycerol (0.2%) and OADC supplement (Merck, Germany). The cultures were incubated at 37°C for 2–3 months without shaking. Bacterial cells were pelleted by centrifugation (4150 g, 10 min) before the supernatant was collected and filtrated (0.2 µm filter). For control purposes not inoculated broth was sterile filtrated and used in experiments.

### RNA-Sequencing and Gene Expression Analysis

For each MDM subgroup, 200 homogenous cells were pooled for RNA extraction, reverse transcribed and amplified with QIAseq FX Single Cell RNA Library Kit (Qiagen, Gemany). Libraries were built for the cDNA generated from each group and sequenced using Illumina X Ten platform with paired-end 150 bp read length. Reads were filtered for the ones with low quality and adapters were mapped to the human reference genome hg19 (GRCh37) with STAR 2.6 (20). Raw reads were counted for each uniquely mapped gene with featureCounts (21). With a negative binomial distribution-based model adopted by edgeR, the gene expression levels were normalized among replicates and between groups, and logarithmic transformation of normalized count per million (logCPM) for each gene was used for further comparative analysis. Gene expression was compared between groups using edgeR with quasi-likelihood F-tests (22). The significance level was preset as a multi-testing corrected False Discovery Rate (FDR) of 0.05. Sequencing data can be found at GenBank, accession number PRJNA817162.

### Incubation of MDM for LM Metabololipidomics by UPLC-MS-MS

Pro-inflammatory M1-like MDM (2×10^6^/ml) were incubated in PBS containing 1 mM CaCl_2_. *M.tb* (H37Rv strain)-conditioned medium (MTB-CM, 1%) or the 19-kD antigen were added for 4 h at 37°C. Signs for apoptotic or cytotoxic effects of MTB-CM or p19 antigen (number and integrity of cells routinely counted after treatment with MTB-CM or 19-kD antigen by light microscopy and trypan blue staining) were not obvious. The supernatants were then transferred to 2 ml of ice-cold methanol containing 10 ml of deuterium-labeled internal standards (200 nM d8-5S-hydroxyeicosatetraenoic acid, d4-leukotriene B_4_, d5-lipoxin A_4_, d5-resolvin D2, d4-PGE_2_ and 10 mM d8–arachidonic acid [AA]) to accomplish quantification and sample recovery. Sample preparation was conducted by adapting published criteria (23, 24). Briefly, samples were kept at -20°C for 60 min to allow protein precipitation. After centrifugation at 1200×g and 4°C for 10 min, 8 ml acidified water was added (final pH = 3.5), and the samples were subjected to solid phase extraction. Solid phase cartridges (Sep-Pak Vac6cc 500 mg/6 ml C18; Waters, Milford, MA) were first equilibrated with 6 ml methanol and 2 ml water prior to sample loading onto columns. After washing with 6 ml water and additional 6 ml *n*-hexane, LM were eluted with 6 ml methyl formate. Finally, the samples were brought to dryness using an evaporation system (TurboVap LV; Biotage, Uppsala, Sweden) and resuspended in 100 µl methanol–water (50/50, v/v) for UPLC–MS-MS automated injections. LM profiling was analyzed with an Acquity UPLC system (Waters) and a QTRAP 5500 Mass Spectrometer (AB Sciex, Darmstadt, Germany) equipped with a Turbo V Source and electrospray ionization. LM were eluted using an Acquity UPLC BEH C18 column (1.7 µm, 2.1 mm x 100 mm; Waters, Eschborn, Germany) at 50°C with a flowrate of 0.3 ml/min and a mobile phase consisting of methanol–water–acetic acid of 42:58:0.01 (v/v/v) that was ramped to 86:14:0.01 (v/v/v) over 12.5 min and then to 98:2:0.01 (v/v/v) for 3 min. The QTrap 5500 was operated in negative ionization mode using scheduled multiple reaction monitoring coupled with information-dependent acquisition. The scheduled multiple reaction monitoring window was 60 s, optimized LM parameters (i.e., collision energy, entrance potential, declustering potential, collision cell exits potential) were adopted (23, 24). The curtain gas pressure was set to 35 psi. The retention time and at least six diagnostic ions for each LM were confirmed by means of external standards (Cayman Chemical/Biomol GmbH, Hamburg, Germany). Quantification was achieved by calibration curves for each LM.

### Western Blot Analysis

Cell lysates of M1-MDM, corresponding to 2 × 10^6^ cells, were separated on 10% polyacrylamide gels, and blotted onto nitrocellulose membranes (Amersham Protran Supported 0.45 μm nitrocellulose, GE Healthcare, Freiburg, Germany). The membranes were then incubated with the following primary antibodies: rabbit polyclonal anti-COX-1, 1:1000 (4841S, Cell Signaling); rabbit polyclonal anti-COX-2, 1:1000 (4842S, Cell Signaling); rabbit monoclonal anti-ERK1/2, 1:1000 (4695S, Cell Signaling); mouse monoclonal anti-phospho-ERK1/2 (Thr202/Tyr204), 1:1000 (9106, Cell Signaling); rabbit monoclonal anti-p38 MAPK, 1:1000 (8690S, Cell Signaling); rabbit polyclonal anti-phospho-p38 MAPK (Thr180/Tyr182), 1:1000 (9211S, Cell Signaling); rabbit monoclonal anti-NF-κB p65 (C22B4), 1:1000 (4764S, Cell Signaling); mouse monoclonal anti-phospho-NF-κB (Ser536), 1:1000 (13346S, Cell Signaling); mouse monoclonal anti-β-actin,1:1000 (3700S, Cell Signaling); rabbit polyclonal anti-β-actin, 1:1000 (4967S, Cell Signaling). Immunoreactive bands were stained with IRDye 800CW Goat anti-Mouse IgG (H+L), 1:10,000 (926-32210, LI-COR Biosciences, Lincoln, NE), IRDye 800CW Goat anti-Rabbit IgG (H+L), 1:15,000 (926-32211, LI-COR Biosciences) and/or IRDye 680LT Goat anti-Mouse IgG (H+L), 1:40,000 (926-68020, LICOR Biosciences), and visualized by an Odyssey infrared imager (LI-COR Biosciences). Data from densitometric analysis were background-corrected.

### Real-Time Quantitative PCR

Extraction of total RNA was performed with the RNAeasy Minikit (Qiagen). The cDNA was synthesized using an oligo-dT primer and SuperScript II reverse transcriptase (Invitrogen, Carlsbad, CA). Gene expression was assessed using SYBR Green-based real-time quantitative PCR as previously described (17). Data were calculated by the 2−ΔΔCT method using β-actin as house-keeping gene.

### Statistical Analysis

Results are given as mean ± S.E.M. of n observations, where n represents the number of experiments with different donors performed on different days as indicated. Analyses of data were performed using GraphPad Prism 8 software (San Diego, CA). Paired t-test was used for comparison of two groups. For multiple comparisons, ANOVA with Bonferroni or Dunnett´s *post hoc* tests were applied as indicated. The criterion for statistically significant is p < 0.05.

## Results

### Sorting and RNA-Sequencing of Human MDM Infected With *M.tb*

**Figure 1:**
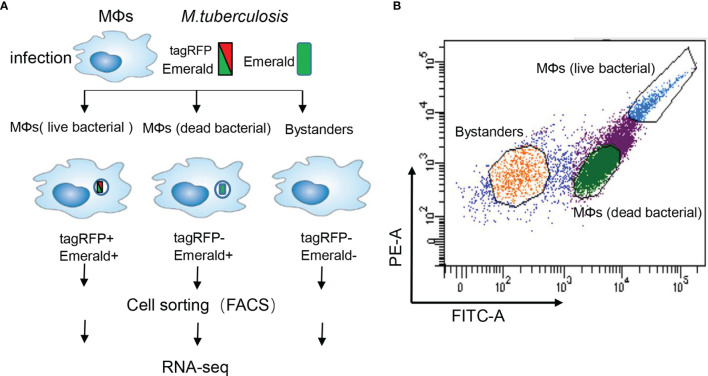
Experimental strategy to sort human MDM with different bacterial content. **(A)** Schematic representation of the workflow. Human monocytes were differentiated with GM-CSF (20 ng/ml) for 6 days towards M0_GM-CSF_MDM (briefly “MDM”). These MDM were then infected with the *M.tb* strain H37Ra (MOI =10) harboring a dual-color reporter that comprises a constitutively (Emerald, green) and an anhydrotetracycline-inducible (tag-RFP, red) fluorescent protein: the constitutive Emerald fluorophore indicating total bacterial burden, and a tagRFP-positive for detection of live bacteria in MDM. After 6 h exposure of MDM, the bacteria were washed away, and MDM were cultured for another 72h. A heterogeneous population of MDM is detectable that consists of cells with live bacteria (left), cells with dead bacteria (middle), and uninfected bystanders (right). **(B)** Representative scatter plots of the infected MDM. The yellow gate captures bystanders, green gate captures MDM with dead bacteria, and blue gate captures MDM with live bacteria.

To characterize outcomes of individual *M.tb*-macrophage interactions, we infected M0_GM-CSF_MDM (briefly “MDM”) with the *M.tb* H37Ra strain carrying an established fluorescence dilution plasmid that reports the bacterial status (live or dead) after internalization. This plasmid provides two different fluorescent protein markers: a constitutively expressed (Emerald, green) and an anhydrotetracycline-inducible (tag-RFP, red) fluorescent protein yielding three distinct outcomes: (1) infection of the MDM with intracellular survival of live *M.tb*, (2) infection resulting in intracellular dead bacteria, and (3) no infection. While MDM with live bacteria display both red and green fluorescence, MDM with dead bacteria display only green fluoresce, and exposed but uninfected MDM display no fluorescence (**Figure 1A**). Importantly, using these reporters it is possible to distinguish MDM that had been initially infected but cleared the bacterium (Emerald+, tagRFP–) from cells that had never been infected (Emerald-, tagRFP–) designated as bystanders. We used fluorescence-activated cell sorting (FACS) to reveal the diverse phenotypes as well as to isolate uninfected MDM and MDM infected with live or dead bacteria (**Figure 1B**). The gating strategy enabled the selective isolation of MDM subgroups confirmed by CFU assays (**Supplementary Figure S1A**).

### Differential Gene Expression Between Live and Dead *M. tb*-Infected MDM

We then subjected 200 isolated MDM of each subgroup to RNA-seq analysis. With the single-cell-like RNA-seq library preparing strategy, the gene expression of few subgroup cells could be well profiled. All of the subgroups in each repeat had more than or nearly 10 million reads that could be uniquely mapped to human genomes (**Supplementary Figure S2A**). The genes covered for each subgroup with > 1 count per million reads (CPM) were around 11,000 and quite even among subgroups or repeats.

**Figure 2:**
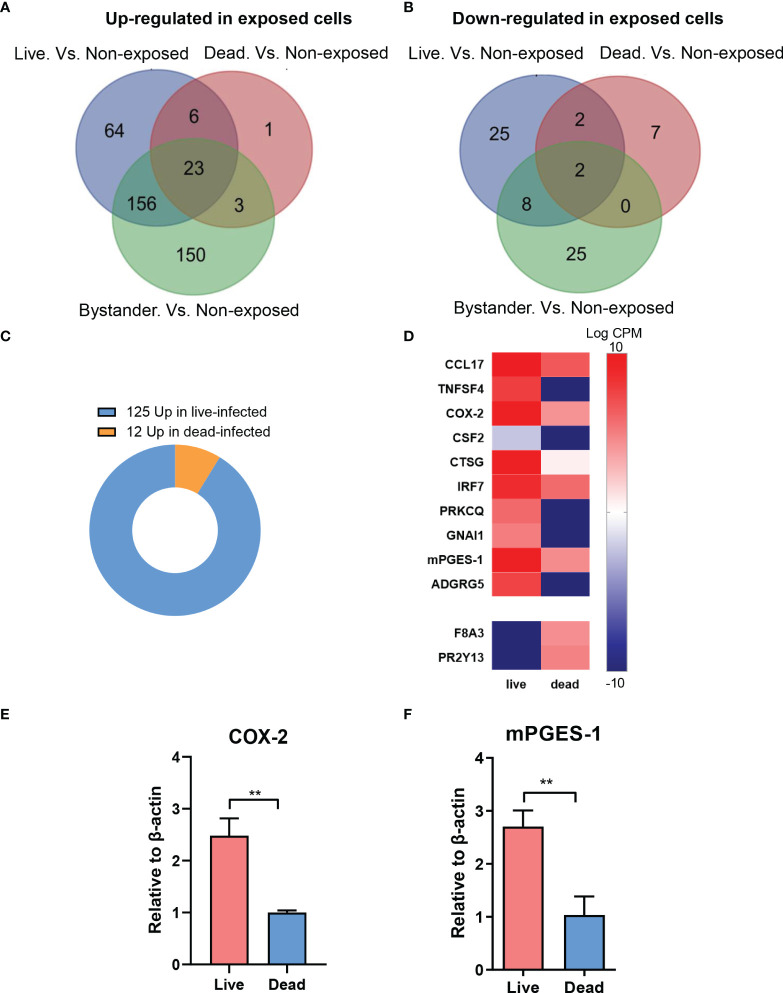
Differential gene expression between live and dead *M.tb*-infected MDM. **(A)** Bystanders, live bacteria-infected and dead bacteria-infected M0_GM-CSF_MDM (MDM) were compared with non-exposed cells, independently. Compared with non-exposed MDM, 332 and 249 genes were detected with higher expression levels in the bystander subgroup and live bacteria-infected subgroup, while 36 and 37 genes were detected with decreased expression, respectively (FDR < 0.05, fold change > 3). **(B)** MDM with dead bacteria showed 33 genes up-regulated and 12 genes down-regulated. In total, 23 genes always showed higher expression while 2 showed decreased levels in bacteria-exposed cells. **(C)** Totally, 137 differentially expressed genes were disclosed between live-infected and dead-infected MDM, with a majority (125/137, 91.2%) up-regulated and only a small subset (12/137, 8.8%) down-regulated in cells with live bacteria. **(D)** Up-regulated genes in MDM with live bacteria versus cells with dead bacteria, mainly related to immune processes, are shown in a heatmap. **(E, F)** Analysis of mRNA levels of COX-2 **(E)** and PTGES **(F)** in live bacteria- and dead bacteria-infected MDM. Relative mRNA levels are normalized to β-actin. Data were log-transformed for statistical analysis by paired t test. **p ≤ 0.01.

MDM bystanders, MDM with live bacteria, and MDM with dead bacteria were compared with a fourth group that are non-exposed MDM, independently. 332 and 249 genes were detected with higher expression levels in the bystander subgroup and the live bacteria subgroup, while 36 and 37 genes showed decreased expression, respectively (FDR < 0.05, fold change > 3). MDM with dead bacteria, however, showed fewer genes expressed with significant differences to non-exposed cells, with only 33 genes being up-regulated and 11 genes down-regulated (**Figure 2A**). 23 genes in individual exposed MDM subgroups versus non-exposed ones showed always higher expression while 2 genes showed decreased levels in bacteria-exposed cells (**Figures 2A, B**).

We next compared the transcriptome profile of MDM exposed to intracellular live and dead bacteria. Totally, 137 genes were differentially expressed between the two subgroups, with a majority (125/137, 91.2%) that were up-regulated and only a small subset (12/137, 8.8%) that were down-regulated in MDM with live bacteria (**Figure 2C**). Many up-regulated genes in MDM with live bacteria are involved in immune response processes including the genes encoding COX-2 and mPGES-1 (PTGES) which are the key enzymes in PGE_2_ biosynthesis (**Figure 2D**). qRT-PCR experiments confirmed higher expression levels of COX-2 and mPGES-1 in MDM with live bacteria (**Figures 2E, F**). These data suggest that secreted factors released from *M.tb* increase the expression pro-inflammatory genes such as COX-2 and mPGES-1 in MDM leading to formation of pro-inflammatory PGE_2_ which might be one mechanism by which bacteria escape intracellular killing during the process of infection.

### *M.tb* Infection of MDM Induces Pro-Inflammatory COX-Derived LM Production and Cytokine Release Which Is Blocked by SASAP

**Figure 3:**
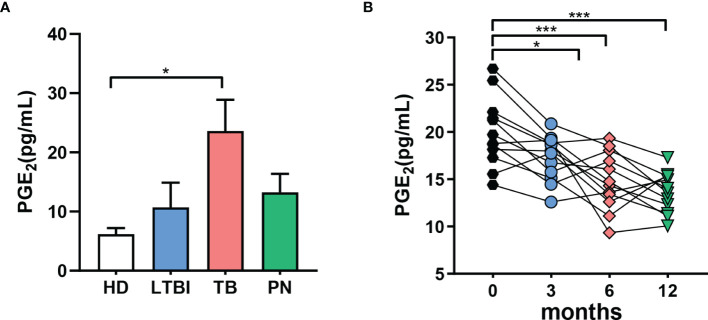
Increased levels of PGE_2_ are associated with active TB in humans. **(A)** Concentrations of PGE_2_ in the plasma of humans determined by ELISA; healthy donors (HD, n = 10), latent TB infection (LTBI, n = 10), pulmonary tuberculosis (TB, n = 10), subjects suffering from non-TB lung disease (non-TB, n = 10). **(B)** PGE_2_ levels in the plasma of active TB patients at the indicated time points after initiation of anti-TB chemotherapy, determined by ELISA; ‘m’ represents months of antibiotic treatment. *p ≤ 0.05, ***p ≤ 0.001.

Based on the results from above, we hypothesized that elevated PGE_2_ in the plasma of TB patients may correlate with the severity of the disease. We analyzed the PGE_2_ plasma levels in patients with either latent TB infection (LTBI), pulmonary tuberculosis (TB) or in patients suffering from non-TB lung disease (non-TB) as well as in healthy donors (HD). The concentration of PGE_2_ in the plasma of TB patients was significantly higher versus healthy cohorts or patients with non-TB lung diseases or latent TB infection (**Figure 3A**). Moreover, plasma PGE levels in active TB patients were assessed for 12 months after initiation of anti-TB chemotherapy. As shown in **Figure 3B**, plasma PGE_2_ levels decreased significantly over the first three months of TB chemotherapy with consistent impairment up to 12 months.

**Figure 4:**
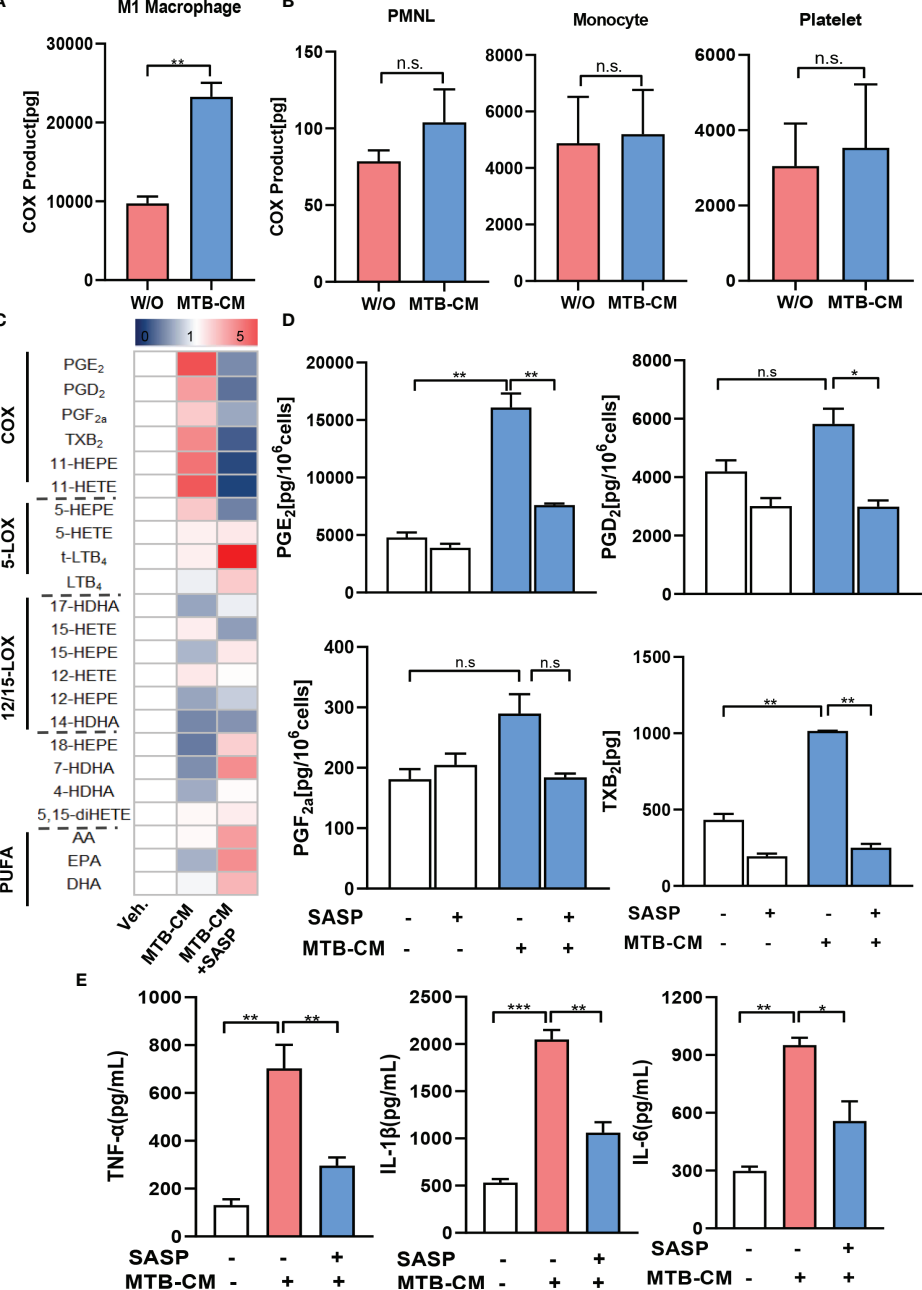
Lipid mediator biosynthesis and cytokine release in M1-MDM exposed to *M.tb*-conditioned medium (MTB-CM). **(A)** Human monocytes were differentiated towards M0_GM-CSF_MDM and then polarized for 48 h with 100 ng/ml LPS plus 20 ng/ml IFN-γ to obtain M1-MDM. **(B)** PMNL, monocytes and platelets were freshly isolated from human blood. Cells were incubated for 4 h with MTB-CM at 37°C and COX products (PGE_2_, PGD_2_, PGF_2_α, TXB_2_) released into the culture medium were extracted and analyzed by UPLC-MS-MS. **(C)** M1-MDM were pretreated with 200 μM SASP or vehicle (veh., 0.1% DMSO) for 1 h and then incubated for 4 h with or without MTB-CM at 37°C. Formed LM were extracted from the supernatants and analyzed by UPLC–MS-MS; detection limit: 0.5 pg. Relative changes of LM given as means, shown in a heatmap, n = 3 separate donors. **(D)** Absolute values of single COX products (PGE_2_, PGD_2_, PGF_2_α, TXB_2_) given in pg/ml; data are means ± S.E.M., n =3. Data were log-transformed for statistical analysis by paired t test. **(E)** Analysis of TNF-α, IL-1β and IL-6 by ELISA given as pg/ml; data are means ± S.E.M., n =3. The differences among groups were compared using one-way ANOVA followed by Tukey’s multiple comparison test *P<0.05, **P < 0.01, ***p ≤ 0.001. n.s., not significant.

It appeared reasonable that the elevated expression of COX-2 and mPGES-1 as well as the corresponding PGE_2_ levels in MDM are caused by secreted factors released from *M.tb*. In fact, several pathogenic bacteria secrete exotoxins or other factors that act on host cells to provoke PGE_2_ and related lipid mediator formation, such as *S. aureus*-derived α-hemolysin (25), lipoteichoic acid (26) or phenol-soluble modulins (27). Several *M.tb* secreted effector molecules are known to modify host cell pathways such as phagosome maturation, cell death, cytokine response, xenophagy, reactive oxygen species response *via* NADPH oxidase 2, nitric oxide response *via* NO synthase 2 and antigen presentation *via* MHC class I and class II molecules (28). Thus, we hypothesized that also for *M.tb* such secreted factors may be causative for the induction of COX-2. We thus studied if *M.tb*-conditioned medium (MTB-CM) would be capable of inducing COX product formation on the cellular level *in vitro* and also addressed other related LM branches such as 5-LOX- and 12/15-LOX-derived products in MDM and for comparison in relevant immune or blood cells. Thus, human M1-MDM and freshly isolated monocytes, PMNL, and platelets from human peripheral blood of healthy volunteers were stimulated with MTB-CM for 4 h and metabololipidomics using UPLC-MS-MS (23, 25) was performed to assess the respective LM signature profiles. Exposure of M1-MDM to MTB-CM caused strong formation of COX-derived prostanoids (PGD_2_, PGE_2_, PGF_2_α, TXB_2_, 11-HETE and 11-HEPE), most prominently PGE_2_ (**Figure 4A**), but not upon exposure of PMNL, monocytes or platelets (**Figure 4B**; **Supplementary Table 1**). Nevertheless, in PMNL, a minor increase in the formation of 5-LOX-derived products (e.g., LTB_4_, t-LTB_4_ and 5-HETE) and other LOX-derived LM like 17-HDHA, 14-HDHA, 12-HETE, 12-HEPE or 4-HDHA was apparent (**Supplementary Table 1**).

We found before that mice treated with SASP had significantly reduced *M.tb* bacterial load in the lung and other organs (17). We hypothesized that SASP-mediated decrease of *M.tb* bacterial loads could be due to interference of the drug with COX-2/mPGES-1 and concomitantly lowered PGE_2_ formation. Thus, we pre-incubated the M1-MDM with 200 µM SASP prior to exposure to MTB-CM and measured LM formation after 4 h. MTT-based cytotoxicity assays indicated no significant decrease of MDM viability by treatment with 200 µM (data not shown), which is in line with result from our previous report (17). The MTB-CM-induced release of PGE_2_ and of other COX-derived prostanoids into the supernatant of M1-MDM was efficiently suppressed by SASP (**Figures 4C, D**), while LM generated by LOX pathways were hardly affected (**Figure 4C**). Note that also the MTB-CM-induced release of pro-inflammatory cytokines such as TNF-a, IL-1β and IL-6 from M1-MDM was significantly reduced by SASP pretreatment (**Figure 4E**).

**Figure 5:**
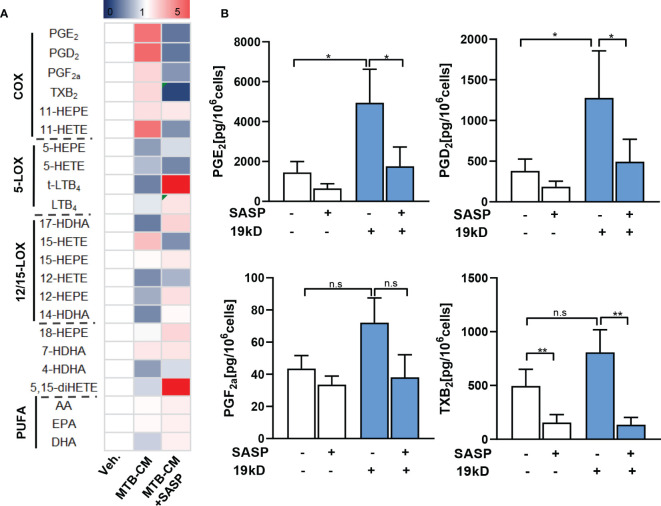
Lipid mediator biosynthesis in M1-MDM exposed to the 19-kD antigen. Human monocytes were differentiated towards M0_GM-CSF_MDM and then polarized for 48 h with 100 ng/ml LPS plus 20 ng/ml IFN-γ to obtain M1-MDM. Cells were then pre-treated with 200 μM SASP or vehicle (veh., 0.1% DMSO) for 1 h and then incubated for 4 h with or without the 19-kD antigen (2 µg/ml) at 37°C. Formed LM were extracted from the supernatants and analyzed by UPLC–MS-MS; detection limit: 0.5 pg. **(A)** Relative changes of LM given as means, shown in a heatmap, n = 3 separate donors. **(B)** Absolute values of single COX products (PGE_2_, PGD_2_, PGF_2_α, TXB_2_) given in pg/ml; data are means ± S.E.M., n =3. Data were log-transformed for statistical analysis by paired t test; *p ≤ 0.05, **p ≤ 0.01. n.s., not significant.

The 19-kD antigen was previously identified as a major virulence factor of *M.tb* (29) and it appeared possible that this antigen is, at least in part, causative for the MTB-CM-induced COX product formation in MDM. We thus exposed the M1-MDM to the isolated 19-kD antigen (2 µg/ml) and analyzed LM formation after 4 h. In line with the results obtained from M1-MDM stimulated by MTB-CM, high levels of COX products, with PGE_2_ as most abundant LM, were evident in the supernatants of 19-kD antigen-activated M1-MDM, and this was clearly reduced when cells were pretreated with SASP (**Figures 5A, B**). In contrast, the levels of PGE_2_ and other COX products were not elevated when M1-MDM were exposed to the early secretory antigenic target 6 (ESAT6) (**Supplementary Table 4**), which is another important virulence factor of *M.tb* (30). Together, MTB-CM and the 19-kD antigen induce formation of PGE_2_ and other COX products in M1-MDM, which is efficiently blocked by SASP, with minor effects on LOX-derived LM formation.

### Reduced COX-2 Expression by SASP Correlates With Impaired NF-κB Activation

**Figure 6:**
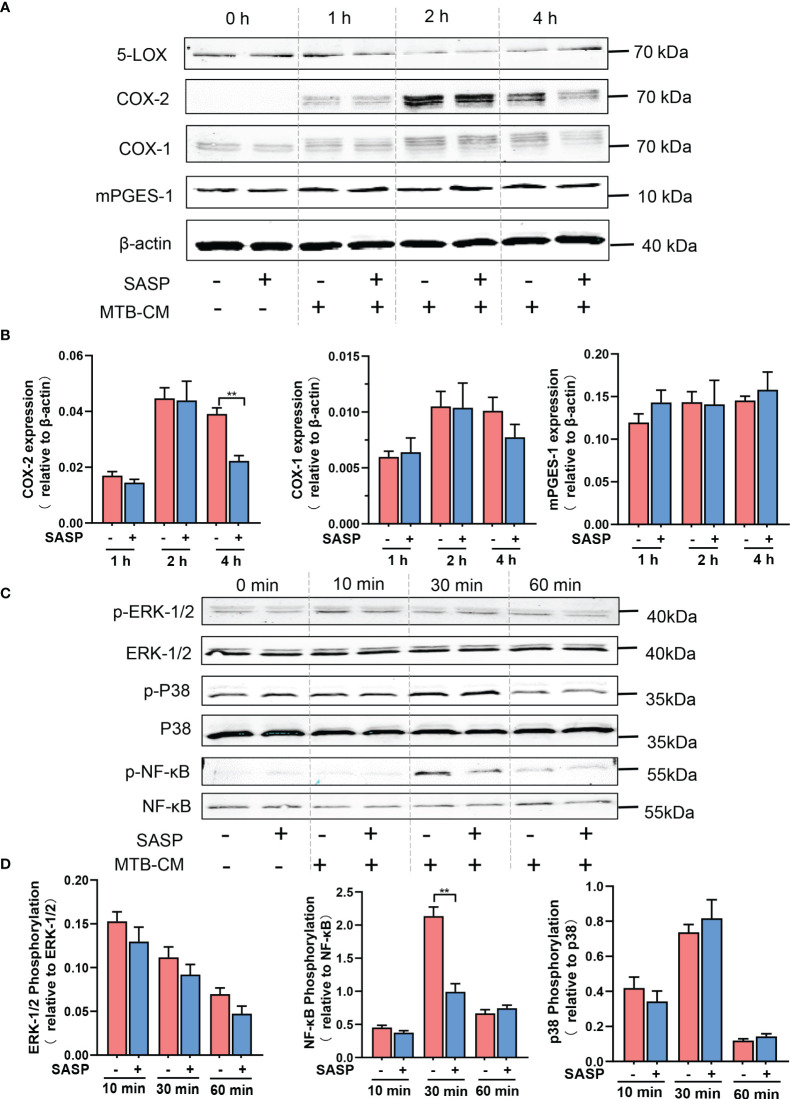
Inhibition of COX-2 protein expression by SASP correlates with impaired phosphorylation of NF-κB p65. **(A, B)** Human M1-MDM were pretreated with 200 μM SASP or vehicle (veh., 0.1% DMSO) for 1 h before stimulation with MTB-CM (1%) for 0, 1, 2 or 4h. Cell lysates were immunoblotted for COX-1, COX-2, and mPGES-1 and normalized to β-actin for densitometric analysis. Data are given as means ± S.E.M, n =3. *p ≤ 0.05, **p ≤ 0.01. **(C, D)** Human M1-MDM were pretreated with 200 μM SASP or vehicle (veh., 0.1% DMSO) for 1 h before stimulation with MTB-CM (1%) for 0, 10, 30 or 60 min. Cell lysates were immunoblotted for phospho-p38 MAPK, p38 MAPK, phospho-ERK-1/2, ERK-1/2, phospho-NF-κB p65, and NF-κB p65, and subjected to densitometric analysis, where signals of phosphorylated proteins were normalized to respective total amounts. Data are given as means ± S.E.M, n = 3. **p < 0.01.

Our results prompted us to investigate if secreted factors from *M.tb* would increase the expression of PGE_2_-biosynthetic enzymes in human M1-MDM and if this is counteracted by SASP. First, we found that in M1-MDM the expression of COX-2 protein was time-dependently induced by MTB-CM treatment, which peaked after 2 h and decreased again after 4 h (**Figures 6A, B**). Of interest, pretreatment of M1-MDM with SASP impaired COX-2 protein levels after 4 h without effects at earlier (2 h) time points. The expression of COX-1 protein slightly increased after exposure to MTB-CM whereas the level of mPGES-1 did not change. Pre-treatment with SASP did not influence the expression of either COX-1 or mPGES-1 (**Figures 6A, B**).

COX-2 expression in general is governed by different signaling routes and protein kinases including the MKK3/6-p38MAPK, MEK-ERK-1/2, and NF-κB pathways (31–34). We assumed that elevated COX-2 protein levels due to MTB-CM may involve some of these pathways that in turn would be affected by SASP. In M1-MDM exposed to MTB-CM for 30 min, the phosphorylation of NF-κB p65 was significantly increased, while treatment for 10 or 60 min did not alter NF-κB p65 phosphorylation. The phosphorylation of p38MAPK or ERK-1/2 did not change after exposure to MTB-CM. Pre-incubation of M1-MDM with SASP for 1 h and subsequent short-term treatment with MTB-CM for 30 min significantly decreased the phosphorylation of NF-κB p65 (**Figures 6C, D**). These data suggest that the elevated COX-2 expression due to MTB-CM could be conferred through activation of the NF-κB pathway and that SASP may mediate its suppressive effects by blocking NF-κB signaling.

## Discussion

Tuberculosis is transmitted through inhalation of *M.tb*-containing aerosol and in most of the cases, a primary infection is established wherein the bacilli are inhaled. However, active TB can be successfully prevented in individuals by the host immune system, resulting in latent *M.tb* infection (LTBI) with no visible symptoms. This underlines the critical role of the host immune response in controling *M.tb* infection (35, 36). Macrophages are the first line of defense against *M.tb* infections and the interaction of *M.tb* with the macrophage has a major impact on the outcome of infection (37). However, why some macrophages can kill the bacteria, but some macrophages cannot, is incompletely understood. In this study, we established a flow cytometry sorting system which can distinguish dead and alive states of *M.tb* in macrophages. Based on the results of RNAseq, we found that the expression of several genes, particularly of COX-2 and mPGES-1 increased in human MDM with live bacteria. In fact, exposure of MDM to MTB-CM or to the 19-kD antigen strongly increased the expression of the COX-2 protein and the concomitant production of PGE_2_.

The common macrophage responses to bacterial and viral infections is the upregulation of genes related to polarization towards an M1-like phenotype (3, 4). Upon exposure of human M1-MDM to the supernatant of the *M.tb* strain H37Rv and analysis of LM by comprehensive UPLC-MS-MS-based metabololipidomics, only the COX products, including PGE_2_, were increased. The *M.tb* virulent strain H37Rv and the avirulent strain H37Ra are two widely used laboratory reference strains. Although H37Ra and H37Rv show different characteristic as well as genetic variations, the 19-kD antigen is expressed with identical sequence by these two strains. The 19-kD antigen mimicked the effects of the supernatant of the *M.tb* strain H37Rv in human M1-MDM, suggesting that this virulence factor (29) but not ESAT6, another important virulence factor of *M.tb* (30), is causative for induction of the COX-2/mPGES-1 pathway. Interestingly, after treatment with SASP, the level of the COX products decreased to almost the same level as the negative control. This may explain our previous discovery of SASP as beneficial suppressor of *M.tb* infection in mice (17) which clearly reduced PGE_2_ formation in the present study. These results are in line with reports by others showing increased PGE_2_ levels in TB disease (38, 39) and indicate that high levels of PGE_2_ may reduce the bacterial clearance ability of macrophages related to the severe inflammatory processes in these phagocytes. In this respect, our current results show that M1-MDM exposed to MTB-CM produced high levels of TNF-α, IL-1β and IL-6 which was also suppressed by SASP.

SASP belongs to the disease-modifying antirheumatic drugs (DMARDs) and is long known to exert immunosuppressive and anti-inflammatory effects (40). The mode of action is unclear, but one proposed mechanism is the inhibition of PG formation which would account for the local anti-inflammatory effects in the colon where SASP is cleaved by intestinal bacteria into sulfapyridine and 5-aminosalicylic acid. Several bacteria, including *M.tb* are capable of cleaving SASP, and there were no major differences in the degree of activity shown by different bacteria species (41). However, our previous work (17) showed that SASP significantly enhanced the antimicrobial capacity of macrophages against *M.tb*, but the increased antimicrobial activity of macrophages due to SASP was apparently not caused by the metabolic product 5-aminosalicylic acid. Moreover, there is no evidence that SASP has direct effects on *M.tb* viability in macrophages.

All these findings support the notion that excessive PGE_2_ is associated with severe inflammation, persisting tissue damage, and poor bacterial clearance (42). On the other hand, PGE_2_ suppressed the excessive inflammatory immune response of the human host against *M.tb* infection (39). In fact, PGE_2_ has also been suggested to confer protective responses during TB disease. A recurring theme in *M.tb* infections is that PGE_2_ suppresses necrotic cell death in macrophages which results in the promotion of pathogen resistance and host protection (37). Infection of M1-MDM with attenuated *M.tb* strongly induced COX-2 *de novo* protein synthesis leading to PGE_2_ production, which could protect mitochondria from inner-membrane damage and inhibit the production of the anti-inflammatory lipid mediator LXA_4_ (43). This may be because PGE_2_ exerts both pro- and anti-inflammatory effects which depends on the concentration and timing at local sites of infection (44).

While eicosanoids produced by the COX pathway regulate a wide spectrum of processes, the 5-LOX pathway is rather operative during inflammation to promote bronchoconstriction and to foster leukocyte recruitment to sites of damaged tissue (45). Although we clearly detected 5-LOX, COX-2, COX-1, and mPGES-1 in M1-MDM at the protein level, only COX-2 was markedly upregulated by *M.tb*. Of note, the upregulated expression of COX-2 due to *M.tb* decreased when cells were pre-treated with SASP. These results are in line with the levels of PGE_2_ produced by the COX-2 pathway. Moreover, the NF-κB pathway is involved in induction of COX-2 expression in M1-MDM. Our data show that the phosphorylation status of NF-κB p65 clearly correlated with COX-2 protein level, i.e., increased upon *M.tb* infection but then decreased after SASP treatment, implying a clear correlation between the two. Interestingly, the anti-inflammatory natural product curcumin was shown to promote *M.tb* clearance in differentiated THP-1 macrophages and in human alveolar macrophages through blocking of NF-κB activation (46), and pharmacological NF-κB inhibition in human MDM and alveolar macrophages caused killing of intracellular *M.tb*, apparently *via* induction of apoptosis and autophagy (47).

In determining the outcome of initial *M.tb* infection, the balance between pro- and anti-inflammatory responses is important (8). Indeed, a change towards either an excessive pro-inflammatory or strong anti-inflammatory cytokine response might result in failure of bacterial control and the appearance of active disease (48). SASP is an anti-inflammatory drug downregulating PGE_2_ that in turn is thought to be a switch between bacterial clearance and acute disease. Furthermore, our results also showed that M1 macrophages produce high levels of pro-inflammatory TNF-α, IL-1β and IL-6. Indeed, the reduced clearance of *M.tb* was related to the severe inflammation in macrophages. *M.tb* secretes factors, including the 19-kD antigen, that activate Toll-like receptors (TLR, especially TLR-2) that play critical roles in immune responses upon *M.tb* infection, connected to NF-κB signaling and pro-inflammatory cytokine release (49). SASP blocked NF-κB-mediated cytokine secretion induced by TLR agonists in SW620 colon cells (50) as well as in patients with ulcerative colitis (51). Therefore, it is likely that SASP acts as inhibitor of the TLR/NF-κB/COX-2/mPGES-1 pathway in *M.tb*-challenged macrophages and thereby prevents severe inflammation caused by excessive PGE_2_ during *M.tb* infection.

A variety of steps in the pathogenesis of TB might be potential targets for host-directed therapy such as targeting the granuloma formation, autophagy and cell-mediated immunity (52, 53). In our study, we focused on targeting the inflammatory response by interference with PGE_2_ biosynthetic pathways using SASP. Together, our results suggest that COX-2 might be a novel target for treatment of TB and that SASP might be a potential therapeutic drug which prevents severe inflammation caused by excessive PGE_2_ production in the pathogenesis process of TB.

## Data Availability Statement

The original contributions presented in the study are included in the article/**Supplementary Material**. Further inquiries can be directed to the corresponding authors.

## Ethics Statement

The studies involving human participants were reviewed and approved by Ethical committee of Shenzhen Third People’s Hospital (Shenzshen, China); Ethical committee of the University Hospital Jena (Jena, Germany). The patients/participants provided their written informed consent to participate in this study.

## Author Contributions

WW, SP, XC and OW conceived and designed this study. WW, YN and KZ carried out UPLC-MS-MS, Western Blot and determination of cytokine levels. YW contributed analysis tools. GD collected the samples, SB and CM provided the supernatants of *M.tb*. YD and YC carried out flow cytometry and FACS analysis. WW, CM, XC, OW participated in the manuscript writing. All the authors have reviewed the manuscript. All authors contributed to the article and approved the submitted version.

## Funding

This work was supported by the National Natural Science Foundation (grant number 82070016), the Science and Technology Project of Shenzhen (grant number JCYJ20180507182049853), and by the Deutsche Forschungsgemeinschaft (DFG), Collaborative Research Center SFB 1127 “ChemBioSys” (project number 239748522, project A04).

## Conflict of Interest

The authors declare that the research presented was conducted in the absence of commercial or financial relationships that could be construed as a potential conflict of interest.

## Publisher’s Note

All claims expressed in this article are solely those of the authors and do not necessarily represent those of their affiliated organizations, or those of the publisher, the editors and the reviewers. Any product that may be evaluated in this article, or claim that may be made by its manufacturer, is not guaranteed or endorsed by the publisher.
