# Supplementary material for: Mycobacterium tuberculosis-Induced Upregulation of the COX-2/mPGES-1 Pathway in Human Macrophages Is Abrogated by Sulfasalazine
Source: Front Immunol. 2022 May 19;13:849583. doi: 10.3389/fimmu.2022.849583 (PMC9160237; doi:10.3389/fimmu.2022.849583)
Supplement: Supplementary file 1 [file DataSheet_1.docx]

Supplementary Material

## Supplementary Figure 1

##
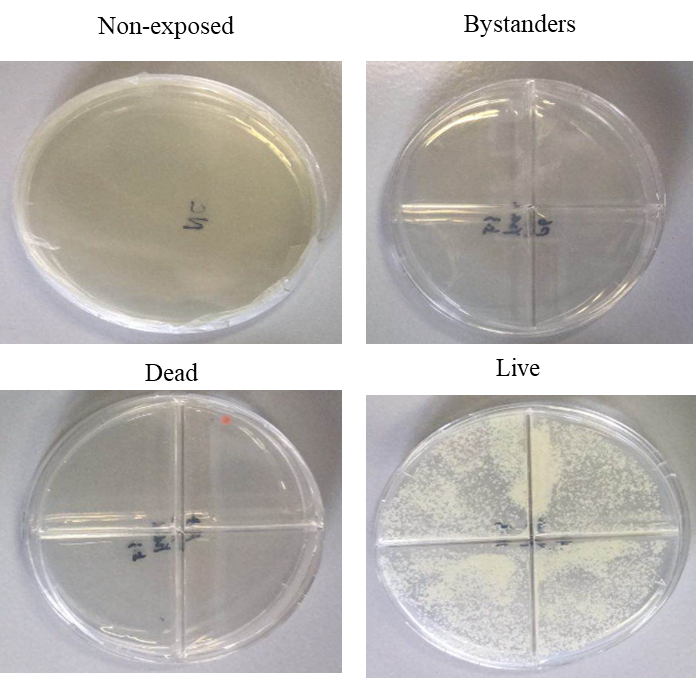


## Supplementary Figure 1. CFU assay for the isolated MDM subgroups. Human monocytes were differentiated by GM-CSF (20 ng/ml) for 6 d to get MGM-CSFMDM. These MDM were infected with the M.tb strain H37Ra harboring a dual-color reporter that comprises a constitutively (Emerald, green) and an anhydrotetracycline-inducible (tag-RFP, red) fluorescent protein, the constitutive Emerald fluorophore indicating total bacterial burden and a tagRFP-positive indicating live bacteria in the MDM. After 6 h incubation of MDM with bacterial MOI = 10, the bacteria were washed away, and cells were cultured for another 72 h. Fluorescence-activated cell sorting (FACS) was used to isolate uninfected cells and cells infected with live or dead bacteria; 200 cells were isolated in each subgroup to perform the CFU assay.

## Supplementary Figure 2


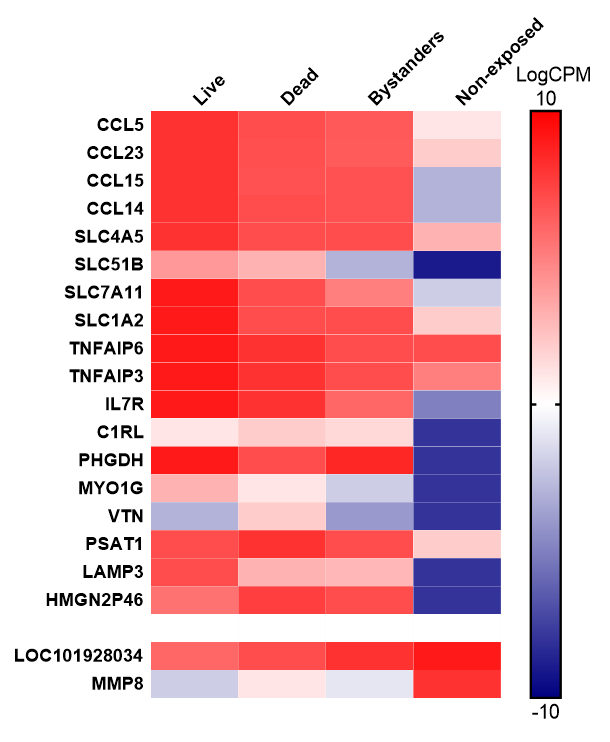


**Supplementary Figure 2.** Heat map for the Gene expression in M. tuberculosis-exposed and -non-exposed MDM. Bystanders, live bacteria-infected and dead bacteria-infected M0GM-CSFMDM (“MDM”) were compared with non-exposed cells independently. Alterations of expression of genes involved in metabolic process and immune system process are presented in a heat map.

**Supplementary Table 1**


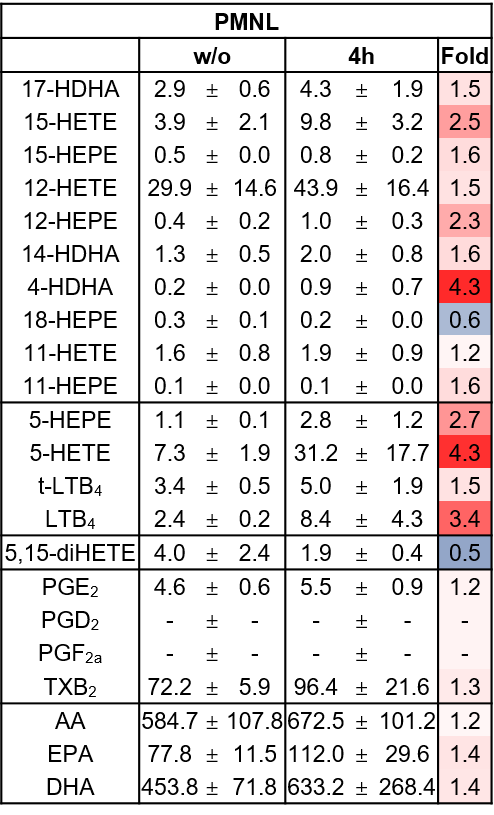

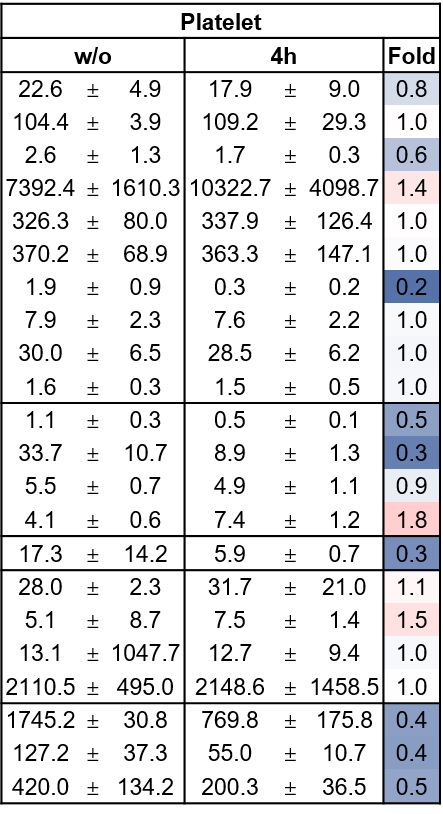

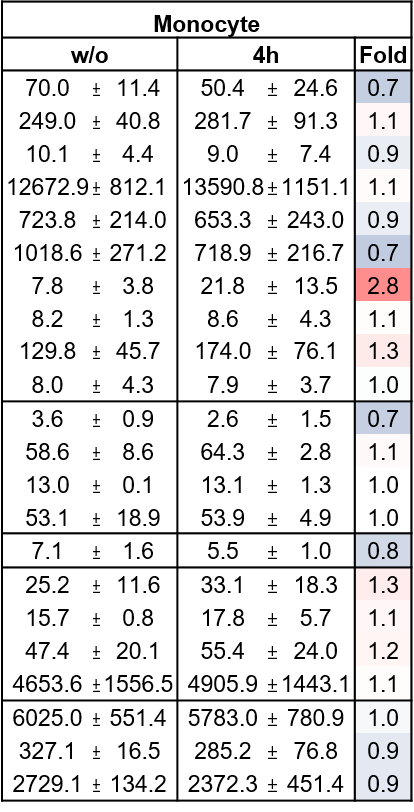


mono-hydroxy

COX

5-LOX

PUFA

**Supplementary Table 1. Lipid mediator biosynthesis in human cells exposed to MTB-CM.** PMNL, monocytes and platelets were incubated for 4 h with the M.tb strain H37Rv-culture medium (MTB-CM) at 37°C. Biosynthesized LM were isolated from the supernatants of the human cell incubations by SPE and analyzed by UPLC–MS-MS; detection limit: 0.5 pg.

**Supplementary Table2**


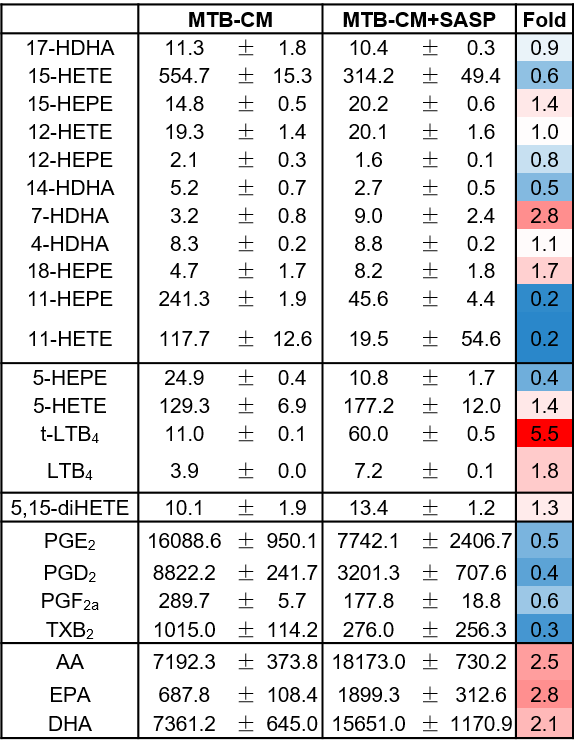

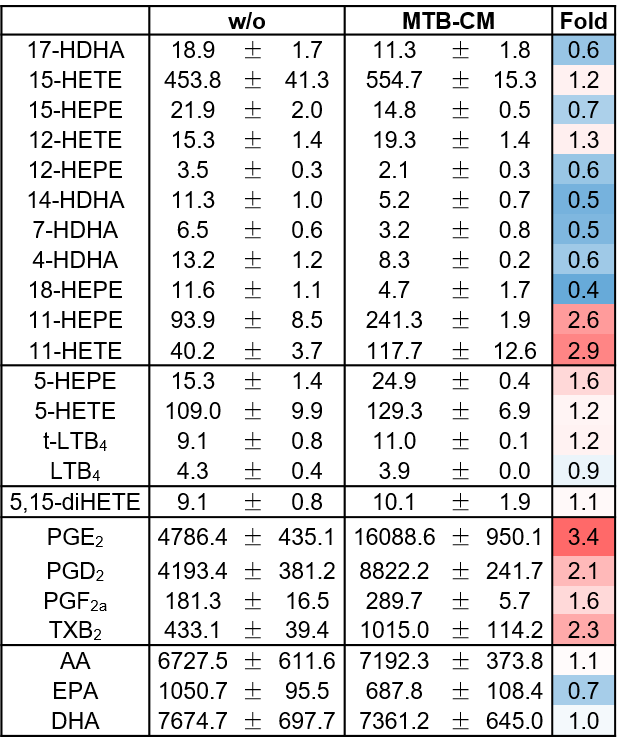


mono-hydroxy

COX

5-LOX

PUFA

mono-hydroxy

COX

5-LOX

PUFA

**Supplementary Table 2. Lipid mediator biosynthesis in M1-MDM exposed to MTB-CM.** Human monocytes were differentiated by GM-CSF (20 ng/ml) for 6 d to get M0GM-CSF. These MDM were polarized for 48 h with 100 ng/ml LPS plus 20 ng/ml IFN-γ to get M1-MDM. After pretreatment with 200 μM SASP or vehicle (veh., 0.1% DMSO) for 1 h, cells were incubated for another 4 h with the M.tb strain H37Rv-culture medium (MTB-CM) at 37°C. Biosynthesized LM were isolated from the supernatants by SPE and analyzed by UPLC–MS-MS; detection limit: 0.5 pg. Results are given as means ± SEM (n=3 separate donors).

**Supplementary Table3**


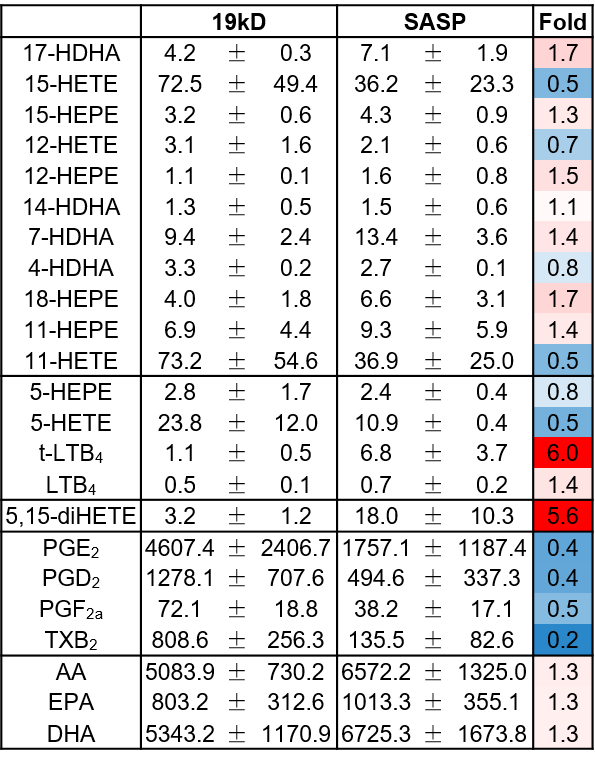

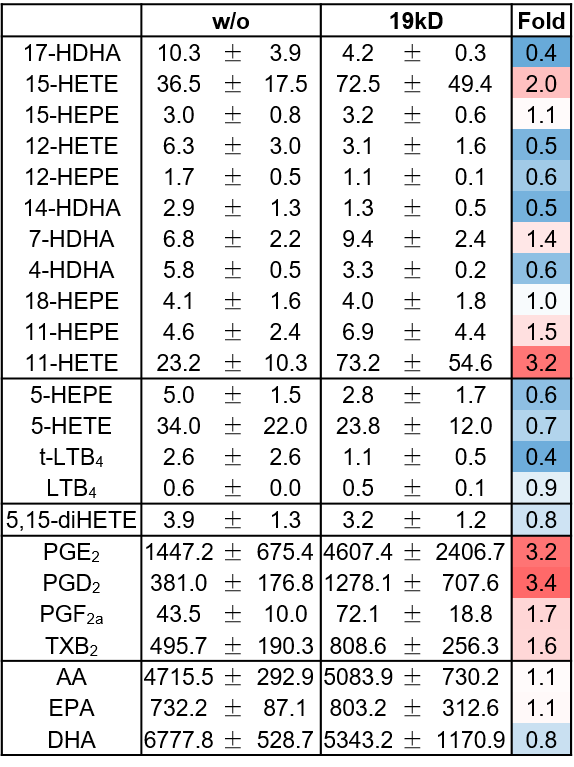


mono-hydroxy

COX

5-LOX

PUFA

mono-hydroxy

COX

5-LOX

PUFA

**Supplementary Table 3. Lipid mediator biosynthesis in M1-MDM exposed to the 19-kD antigen.** Human monocytes were differentiated by GM-CSF (20 ng/ml) for 6 d to get M0GM-CSF. These MDM were then polarized for 48 h with 100 ng/ml LPS plus 20 ng/ml IFN-γ to get M1-MDM. After pretreatment with 200 μM SASP or vehicle (veh., 0.1% DMSO) for 1 h, the cells were incubated for another 4 h with the 19-kD antigen at 37°C. Biosynthesized LM were isolated from the supernatants by SPE and analyzed by UPLC–MS-MS; detection limit: 0.5 pg. Results are given as means ± SEM (n=3 separate donors).


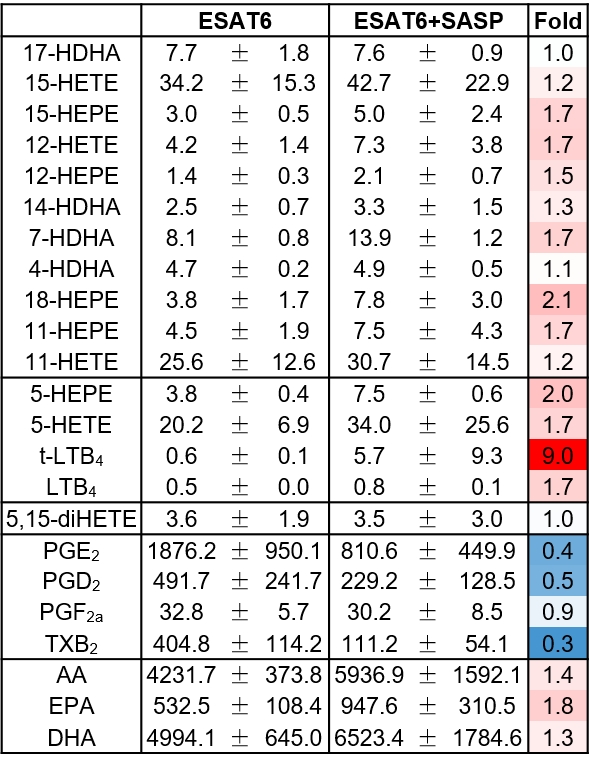
**Supplementary Table 4**


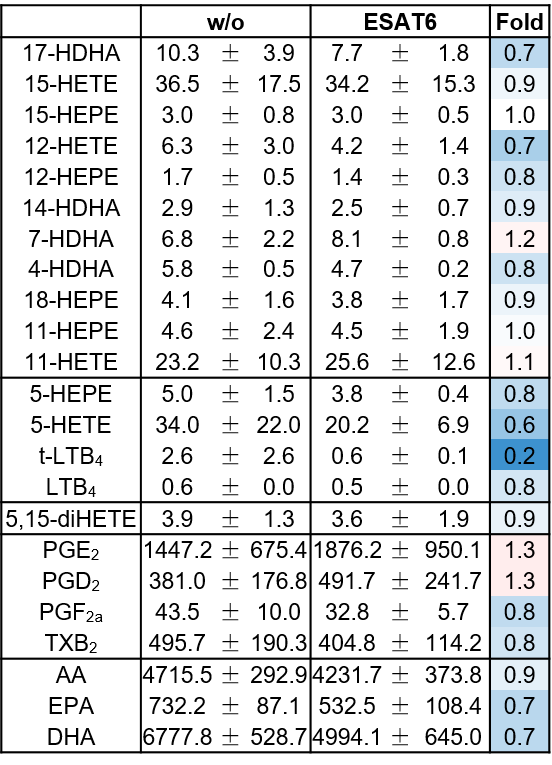


mono-hydroxy

COX

5-LOX

PUFA

mono-hydroxy

COX

5-LOX

PUFA

**Supplementary Table 4. Lipid mediator biosynthesis in M1-MDM exposed to ESAT6.** Human monocytes were differentiated by GM-CSF (20 ng/ml) for 6 d to get M0GM-CSFMDM. These MDM were polarized for 48 h with 100 ng/ml LPS plus 20 ng/ml IFN-γ to get M1-MDM. After pretreatment with 200 μM SASP or vehicle (veh., 0.1% DMSO) for 1 h, the cells were incubated for another 4 h with ESAT6 at 37°C. Biosynthesized LM were isolated from the supernatants by SPE and analyzed by UPLC–MS-MS; detection limit: 0.5 pg. Results are given as means ± SEM (n=3 separate donors).

**Supplementary Figure 3**

**B**

**A**


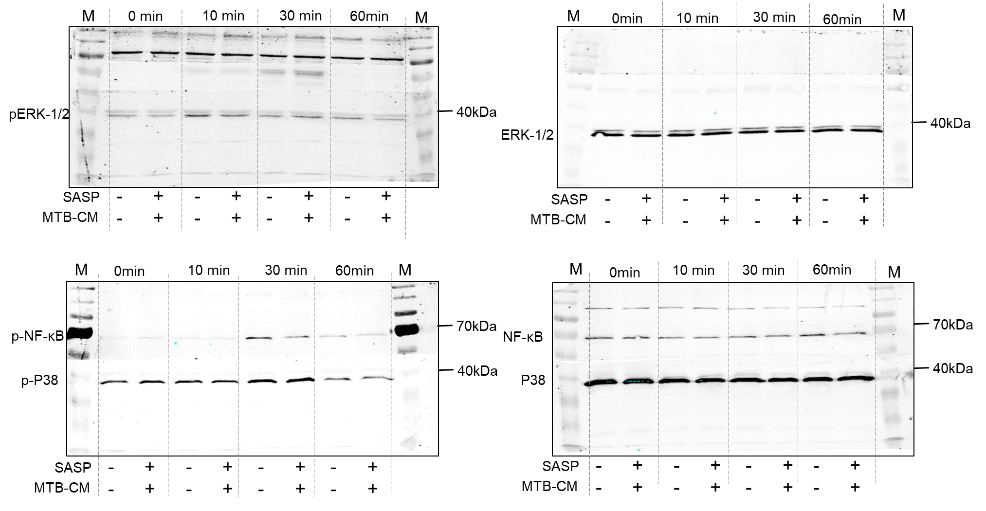

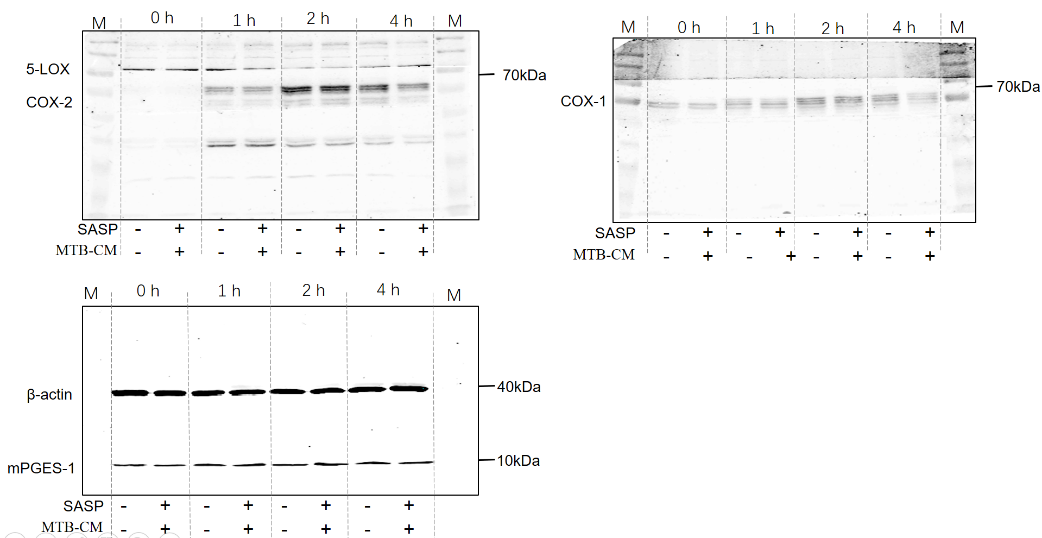


**Supplementary Figure 3. Inhibition of COX-2 protein expression by SASP correlates with impaired phosphorylation of NF-κB p65. (**A) Human M1-MDM were pretreated with 200 μM SASP or vehicle (veh., 0.1% DMSO) for 1 h before stimulation with MTB-CM (1%) for 0, 1, 2 or 4 h. Cell lysates were immunoblotted for COX-1, COX-2, and mPGES-1 and normalized to β-actin . (B) Human M1-MDM were pretreated with 200 μM SASP or vehicle (veh., 0.1% DMSO) for 1 h before stimulation with MTB-CM (1%) for 0, 10, 30 or 60 min. Cell lysates were immunoblotted for phospho-p38 MAPK, p38 MAPK, phospho-ERK-1/2, ERK-1/2, phospho-NF-κB p65, and NF-κB p65.
